# Molecular Targeting of EGFR, BRAF, and HER2 Signaling in Colorectal Cancer: Contemporary Advances with Panitumumab, Encorafenib, and Tucatinib

**DOI:** 10.3390/jcm15062387

**Published:** 2026-03-20

**Authors:** Piotr Kawczak, Tomasz Bączek

**Affiliations:** 1 Department of Pharmaceutical Chemistry, Faculty of Pharmacy, Medical University of Gdańsk, 80-416 Gdańsk, Poland; tomasz.baczek@gumed.edu.pl; 2 Department of Nursing and Medical Rescue, Institute of Health Sciences, Pomeranian University in Słupsk, 76-200 Słupsk, Poland

**Keywords:** colorectal cancer, EGFR, BRAF, HER2, panitumumab, encorafenib, tucatinib, targeted therapy, precision oncology

## Abstract

Metastatic colorectal cancer (mCRC) remains a major cause of cancer-related mortality worldwide. Advances in molecular profiling have transformed the therapeutic landscape, enabling biomarker-driven treatment strategies based on alterations in RAS, BRAF V600E, HER2 amplification, and mismatch repair status. Among these, dysregulation of the epidermal growth factor receptor (EGFR), BRAF, and HER2 signaling pathways represents a central driver of tumor progression and therapeutic resistance. Targeted agents directed against these pathways—including the anti-EGFR monoclonal antibody panitumumab, the selective BRAF inhibitor encorafenib, and the HER2-selective tyrosine kinase inhibitor tucatinib—have substantially expanded treatment options for molecularly defined subgroups of patients with mCRC. Anti-EGFR therapy remains a cornerstone of treatment for patients with RAS/BRAF wild-type, left-sided tumors. Panitumumab combined with chemotherapy has demonstrated significant improvements in response rates and overall survival compared with anti-angiogenic-based regimens in randomized clinical trials. For tumors harboring BRAF V600E mutations, which are associated with poor prognosis, combination strategies incorporating encorafenib with EGFR blockade have shown clinically meaningful survival benefits and represent an important therapeutic advance. In HER2-amplified colorectal cancer, HER2-targeted therapies have emerged as an effective treatment strategy. Trastuzumab-based combinations and HER2-selective tyrosine kinase inhibitors such as tucatinib have demonstrated durable responses and favorable safety profiles in heavily pretreated patients. This review summarizes current evidence from pivotal phase II and III clinical trials, translational studies, and real-world data evaluating EGFR-, BRAF-, and HER2-directed therapies in colorectal cancer. Particular emphasis is placed on biomarker-guided patient selection, mechanisms of resistance, and emerging combination strategies that continue to refine precision oncology approaches in mCRC.

## 1. Introduction

Colorectal carcinogenesis is driven by recurrent alterations in key oncogenic pathways regulating proliferation, survival, and differentiation [1,2,3,4]. Dysregulation of the EGFR pathway, constitutive activation of RAS–RAF–MEK–ERK (MAPK) signaling, and aberrations within the ERBB receptor family—particularly HER2 (ERBB2)—are central drivers of tumor initiation, progression, and therapeutic resistance [5,6,7,8]. Comprehensive genomic analyses have identified clinically relevant molecular subtypes characterized by actionable alterations in EGFR-related pathways, BRAF V600E mutations, and HER2 amplification [9,10,11,12]. These findings establish colorectal cancer (CRC) as a biologically stratified disease rather than a single histology-defined entity.

This molecular framework has been translated into routine clinical practice through guideline-directed profiling. Current international recommendations require extended RAS (KRAS and NRAS) testing, BRAF mutation analysis, and HER2 assessment in metastatic CRC (mCRC) to guide treatment selection and sequencing [13,14,15,16]. Next-generation sequencing is increasingly used to detect rarer actionable alterations and support precision oncology approaches.

Many of these alterations converge on the MAPK (RAS–RAF–MEK–ERK) and PI3K–AKT signaling pathways, which regulate cellular proliferation and survival. In mCRC, BRAF V600E mutations lead to constitutive MAPK activation, whereas HER2 amplification promotes receptor-mediated signaling that enhances downstream pathway activity. Targeted therapies act at different levels of these networks: anti-EGFR monoclonal antibodies block ligand-dependent receptor activation, BRAF inhibitors suppress constitutively active kinase signaling, and HER2 inhibitors interfere with receptor dimerization and downstream signaling. Because these signaling cascades are well characterized, the following sections focus primarily on their therapeutic implications and mechanisms of resistance rather than detailed pathway biology [17,18].

EGFR targeting remains a cornerstone of therapy for patients with RAS wild-type mCRC. Activating RAS mutations predict lack of benefit—and potential harm—from EGFR inhibition, establishing extended RAS testing as mandatory before therapy [19]. Clinical trials confirmed the efficacy of panitumumab in chemorefractory disease and defined its integration with cytotoxic chemotherapy [20]. Randomized studies further demonstrated that EGFR-targeted therapy provides particular benefit in left-sided, RAS wild-type tumors in the first-line setting, refining patient selection by molecular and clinicopathologic criteria [21,22].

BRAF V600E–mutant CRC represents a biologically distinct subtype with aggressive clinical behavior and historically poor outcomes with conventional chemotherapy. Preclinical and translational studies demonstrated that resistance to BRAF inhibitor monotherapy in CRC is mediated by rapid feedback activation of EGFR signaling, in contrast to melanoma [23]. This insight led to combination strategies incorporating BRAF and EGFR inhibition. The BEACON CRC trial established encorafenib plus cetuximab as the standard of care in previously treated BRAF V600E–mutant mCRC, improving overall survival (OS) and response rates compared with standard chemotherapy. Although the addition of binimetinib increased response rates, it did not improve OS and added toxicity; therefore, it is not considered standard of care [24,25]. Ongoing trials are evaluating encorafenib-based combinations earlier in the disease course, reflecting a shift toward earlier biology-driven intervention in high-risk CRC [26,27].

HER2 amplification and overexpression have emerged as actionable drivers in a subset of CRC, particularly in RAS wild-type tumors, where HER2 signaling also mediates resistance to EGFR-targeted therapy [18,28]. Early studies demonstrated the feasibility and activity of dual HER2 blockade with trastuzumab-based combinations in heavily pretreated HER2-positive mCRC [29,30]. Subsequently, tucatinib, a highly selective HER2 tyrosine kinase inhibitor, combined with trastuzumab, showed clinically meaningful efficacy in chemotherapy-refractory, HER2-positive, RAS wild-type mCRC, leading to regulatory approval and integration into treatment algorithms [31,32,33].

Advances in precision diagnostics have further refined CRC management. Consensus molecular subtyping highlights substantial biological heterogeneity with prognostic and therapeutic implications [7,34,35]. Circulating tumor DNA (ctDNA) analysis enables detection of minimal residual disease, prediction of recurrence, and dynamic monitoring of treatment response and resistance [36,37,38]. Tumors with microsatellite instability–high (MSI-H) or mismatch repair deficiency (dMMR) derive substantial benefit from immune checkpoint inhibitors and should be identified early in treatment planning [39]. Because immunotherapy represents a distinct therapeutic paradigm, its detailed clinical application lies beyond the primary scope of this review, which focuses specifically on EGFR-, BRAF-, and HER2-targeted strategies. Rare actionable alterations, including NTRK fusions and POLE mutations, also warrant testing given the availability of effective targeted therapies [40]. In the context of EGFR-directed therapy, ctDNA analyses have demonstrated the emergence of acquired RAS and EGFR alterations under treatment pressure, enabling real-time assessment of resistance and supporting adaptive strategies such as rechallenge [41,42,43]. Collectively, these developments support an iterative, biomarker-guided treatment paradigm in CRC [44,45,46].

This narrative review summarizes contemporary advances in molecularly targeted therapies directed at the EGFR, BRAF, and HER2 pathways in CRC, with emphasis on panitumumab, encorafenib, and tucatinib as representative agents. Particular attention is given to molecular diagnostics, rational combination strategies, and emerging biomarkers to optimize outcomes in CRC.

A structured literature search was conducted in PubMed and Scopus to identify relevant English-language publications published between January 2006 and January 2026. The starting date was selected to capture early translational investigations of EGFR-targeted therapy in CRC, preceding the regulatory approvals of anti-EGFR monoclonal antibodies and the emergence of biomarker-driven treatment strategies. Search terms included combinations of the following keywords: “colorectal cancer”, “metastatic colorectal cancer”, “panitumumab”, “encorafenib”, “BRAF V600E”, “tucatinib”, “HER2”, “EGFR”, “targeted therapy”, and “rechallenge strategies”. Reference lists of relevant articles and major clinical trial publications were also manually screened to identify additional eligible studies. The initial search identified approximately 450 publications. After title and abstract screening for relevance to targeted treatment strategies in mCRC, approximately 350 articles were selected for full-text evaluation. Ultimately, about 260 studies were included in the final narrative synthesis. Eligible studies comprised phase II and III prospective trials, randomized controlled trials, registration studies, meta-analyses, and practice-changing prospective cohort studies. Priority was given to randomized and registration-directed trials that established the clinical roles of anti-EGFR therapy, BRAF-targeted combinations, and HER2-directed treatment strategies. Large real-world cohorts and retrospective analyses were also included when they provided clinically meaningful insights into treatment effectiveness, resistance mechanisms, or therapeutic sequencing in routine practice. Smaller mechanistic or translational studies were incorporated selectively when they contributed to the biological understanding of EGFR, BRAF, or HER2 signaling pathways. Case reports, non-English publications, and conference abstracts without sufficient methodological detail were excluded. Preclinical-only studies were generally excluded unless they provided important mechanistic insights relevant to treatment efficacy, resistance mechanisms, or biomarker-driven therapeutic strategies. In addition, seminal studies published before 2006 were included when necessary to contextualize the biological rationale for targeted therapies discussed in this review. As this review was designed as a narrative synthesis, study selection emphasized clinical relevance, methodological robustness, and impact on contemporary therapeutic practice, rather than formal systematic review criteria.

**Figure 1:**
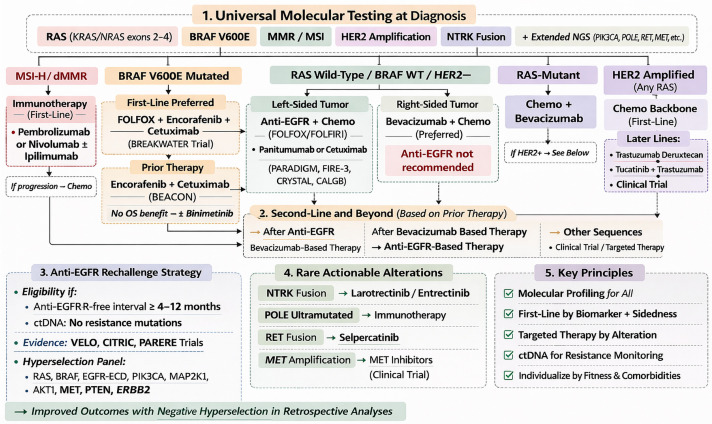
Therapeutic algorithm for metastatic colorectal cancer. The figure presents a conceptual treatment algorithm synthesized by the authors based on current clinical evidence, major clinical trials, and international guideline recommendations. The scheme is an original graphical representation and does not reproduce or replicate any specific published guideline or source. All abbreviations employed are defined in the text in the Abbreviations section.

Figure 1 presents a therapeutic algorithm for mCRC based on contemporary European Society for Medical Oncology (ESMO) 2023 and National Comprehensive Cancer Network (NCCN) 2024 guidelines and recommendations.

## 2. Panitumumab

Panitumumab is a fully human IgG2 monoclonal antibody targeting EGFR and represents a key targeted therapy in colorectal malignancies, particularly CRC [47,48,49]. EGFR is a transmembrane receptor tyrosine kinase within the ErbB family that is frequently overexpressed or dysregulated in CRC. Aberrant EGFR signaling promotes sustained proliferation, resistance to apoptosis, angiogenesis, invasion, and metastatic spread [50,51]. Given the epithelial origin of CRC and the dependence of many tumors on EGFR-driven signaling, inhibition of this pathway is both biologically rational and clinically validated [52].

**Figure 2:**
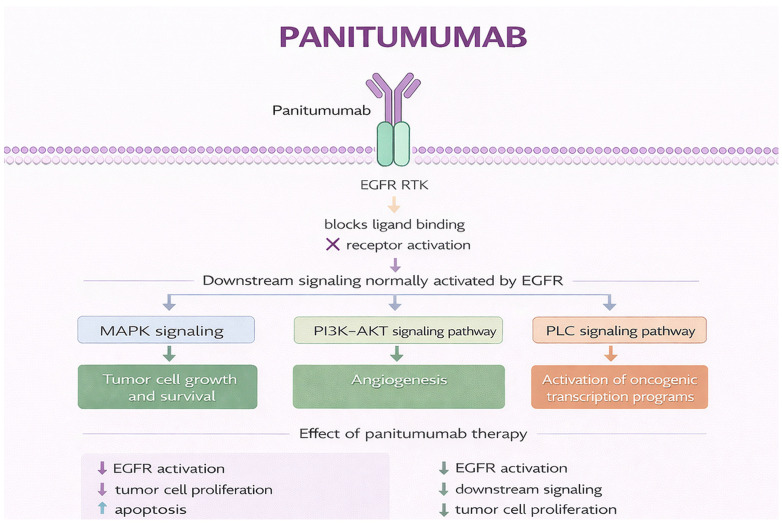
Mechanism of action of panitumumab. Original schematic illustration created by the authors based on the mechanism described in [58]. All abbreviations employed are defined in the text in the Abbreviations section.

Panitumumab binds the extracellular ligand-binding domain of EGFR, preventing activation by endogenous ligands such as epidermal growth factor (EGF) and transforming growth factor-α (TGF-α). This blocks receptor dimerization and autophosphorylation, suppressing downstream signaling through the RAS–RAF–MEK–ERK (MAPK) and PI3K–AKT pathways that regulate proliferation, survival, metabolism, and apoptosis [53,54]. As an IgG2 antibody, panitumumab mediates minimal antibody-dependent cellular cytotoxicity (ADCC); its antitumor activity derives primarily from direct inhibition of signal transduction, distinguishing it mechanistically from IgG1 antibodies such as cetuximab [55,56,57]. Figure 2 illustrates its mechanism of action.

The clinical development of panitumumab paralleled the emergence of biomarker-driven treatment strategies in metastatic CRC (mCRC). Early randomized trials in chemotherapy-refractory disease demonstrated improved progression-free survival (PFS) compared with best supportive care, confirming the therapeutic relevance of EGFR blockade in advanced CRC [59,60,61]. Subsequent translational analyses identified activating KRAS and NRAS mutations as mechanisms of primary resistance, establishing tumor genotype—not EGFR expression—as the key determinant of response [62]. These findings led to regulatory label changes and updates in ESMO and NCCN guidelines mandating extended RAS testing before anti-EGFR therapy [63,64,65].

Randomized trials have since clarified the clinical contexts in which EGFR inhibition provides the greatest benefit. In extended RAS wild-type tumors, anti-EGFR antibodies combined with chemotherapy improve response rates and survival compared with chemotherapy alone [66,67,68]. However, interpretation of these results requires consideration of differences in study design and patient populations. Further analyses demonstrated that primary tumor sidedness is a critical predictive factor. In left-sided RAS wild-type, BRAF wild-type, HER2-negative mCRC, anti-EGFR therapy combined with chemotherapy is generally preferred in the first-line setting, supported by trials including CRYSTAL, FIRE-3, CALGB/SWOG 80405, and PARADIGM [69,70,71,72,73,74]. PARADIGM demonstrated improved OS with panitumumab plus FOLFOX compared with bevacizumab plus FOLFOX in left-sided tumors [21]. In contrast, bevacizumab-based regimens are typically favored in right-sided disease due to inferior outcomes observed with anti-EGFR therapy [75].

Comparative interpretation of key trials highlights important methodological considerations. FIRE-3 suggested an OS advantage for cetuximab over bevacizumab despite similar PFS [76], whereas CALGB/SWOG 80405 reported comparable survival between strategies overall [71]. Differences in chemotherapy backbone, patient selection, molecular testing depth, statistical power, and post-progression therapies likely contributed to these divergent findings. The phase II PEAK study also suggested longer OS with panitumumab compared with bevacizumab in extended RAS wild-type populations, particularly in left-sided tumors [77]. However, the relatively small sample size and phase II design limit definitive conclusions and highlight the need for cautious interpretation. Collectively, these studies support preferential use of anti-EGFR therapy in biologically selected left-sided disease while underscoring the limitations of cross-trial comparisons.

Beyond first-line therapy, panitumumab remains active across later treatment lines in appropriately selected patients. In the second-line setting, panitumumab combined with FOLFIRI improves PFS in RAS wild-type disease [78,79]. In chemotherapy-refractory settings, the ASPECCT trial demonstrated non-inferiority of panitumumab compared with cetuximab monotherapy, confirming comparable efficacy of the two anti-EGFR antibodies [20,80]. Although these results support the continued role of EGFR inhibition in later treatment lines, it should be noted that variations in patient selection and prior therapies across studies may influence observed outcomes.

Anti-EGFR rechallenge has emerged as a potential strategy after a treatment-free interval, particularly when ctDNA analysis demonstrates clearance of resistance mutations [81,82,83,84]. Reported response rates in these studies are encouraging; however, most available evidence derives from small, non-randomized trials or retrospective analyses. Such designs introduce the possibility of selection bias, as patients eligible for rechallenge often represent a subgroup with more favorable tumor biology or slower disease progression. Additionally, heterogeneity in ctDNA assay methodologies, sensitivity thresholds, and definitions of molecular clearance complicates comparisons across studies. Resistance mechanisms—including alterations in RAS, BRAF, EGFR ectodomain, PIK3CA, MAP2K1, AKT1, MET, PTEN, and ERBB2—support the concept of “negative hyperselection” to refine patient selection for rechallenge strategies [82,83,84,85]. Prospective randomized trials are therefore required to confirm the clinical utility of ctDNA-guided rechallenge strategies.

The toxicity profile of panitumumab reflects on-target EGFR inhibition in epithelial tissues. Acneiform rash is the most common adverse event and typically occurs early during treatment. Interestingly, dermatologic toxicity correlates with improved outcomes; pooled analyses demonstrate longer PFS and OS in patients with grade ≥ 2 rash, supporting proactive dermatologic management rather than treatment discontinuation [86,87,88,89]. Additional adverse effects include diarrhea, mucositis, fatigue, and electrolyte disturbances such as hypomagnesemia resulting from impaired renal tubular reabsorption [90,91]. Infusion reactions are less common than with chimeric antibodies, although rare severe toxicities such as interstitial lung disease require prompt discontinuation [92].

Despite its established benefit, several limitations remain. Crossover and heterogeneous post-progression therapies in pivotal trials may confound survival analyses, and frail or elderly patients were often underrepresented. The optimal integration of anti-EGFR therapy with immunotherapy in MSI-H/dMMR tumors and the management of acquired resistance remain areas of ongoing investigation [85].

Within contemporary CRC management, panitumumab is firmly established as a precision therapy restricted to RAS wild-type mCRC, with the strongest evidence supporting its use in left-sided primary tumors. Treatment selection should integrate tumor sidedness, molecular profile, toxicity considerations, comorbidities, and therapeutic goals [71,76,77]. Beyond its clinical benefit, panitumumab exemplifies the broader paradigm of biomarker-driven oncology, demonstrating how molecular stratification can optimize outcomes and advance personalized treatment strategies in CRC [93,94,95,96,97,98,99].

Table 1 summarizes treatment-emergent adverse events and management strategies for panitumumab in CRC, and Table 2 outlines major pivotal clinical trials in mCRC.

## 3. Encorafenib

Encorafenib is an orally available, small-molecule selective inhibitor of BRAF kinase developed to target tumors harboring activating BRAF mutations, most commonly V600E. It represents a major therapeutic advance in CRC, where BRAF V600E mutations occur in approximately 8–12% of cases and are associated with aggressive clinical behavior, early metastatic spread, chemotherapy resistance, and poor prognosis [112,113,114,115]. BRAF is a serine–threonine kinase and a central component of the RAS–RAF–MEK–ERK (MAPK) pathway, which regulates proliferation, differentiation, survival, and oncogenic transformation [116,117,118].

In CRC, the BRAF V600E mutation leads to constitutive activation of MAPK signaling independent of upstream receptor tyrosine kinase stimulation, driving uncontrolled tumor growth [119,120,121]. However, the biology of BRAF-mutant CRC differs substantially from that observed in melanoma. In colorectal epithelial cells, high basal expression of EGFR enables rapid compensatory activation of EGFR signaling following BRAF inhibition. ERK suppression releases negative feedback on EGFR, resulting in MAPK pathway reactivation through upstream receptor signaling [122]. In contrast, melanoma cells exhibit relatively low EGFR expression, limiting this feedback mechanism. This biologic distinction explains the limited efficacy of BRAF inhibitor monotherapy in CRC and established the rationale for combined BRAF and EGFR blockade [122,123,124].

**Figure 3:**
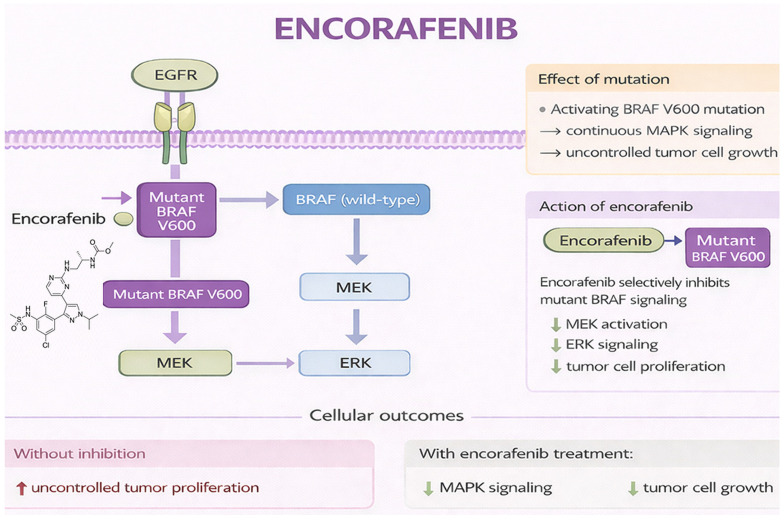
Mechanism of action of encorafenib. Original schematic illustration created by the authors based on the mechanism described in [131]. All abbreviations employed are defined in the text in the Abbreviations section.

Encorafenib inhibits mutant BRAF by binding to the ATP-binding pocket of the kinase, suppressing ERK phosphorylation and downstream oncogenic signaling. Compared with earlier BRAF inhibitors, encorafenib demonstrates higher potency, prolonged target engagement, and a longer dissociation half-life, allowing sustained pathway inhibition with improved tolerability [125,126]. Preclinical and early clinical studies showed that combining BRAF inhibition with EGFR blockade prevents adaptive MAPK reactivation, providing the biological rationale for combination strategies in CRC [127,128,129,130]. Figure 3 illustrates its mechanism of action.

The clinical development of encorafenib therefore focused on dual BRAF–EGFR inhibition strategies. Early clinical studies confirmed that BRAF inhibitor monotherapy is ineffective in CRC due to EGFR-mediated feedback activation, establishing the need for combination therapy and, in some approaches, additional MEK inhibition [122,123,132]. This concept was validated in the phase III BEACON CRC trial, which demonstrated that encorafenib combined with cetuximab significantly improved OS, PFS, and response rates compared with irinotecan-based chemotherapy plus cetuximab in previously treated BRAF V600E–mutant mCRC [133,134,135,136].

Importantly, BEACON showed that the doublet regimen of encorafenib plus cetuximab achieved survival outcomes comparable to the triplet regimen that also included the MEK inhibitor binimetinib. Median OS reached 9.3 months with the doublet compared with 5.9 months in the chemotherapy control arm (hazard ratio 0.61), whereas the triplet regimen increased response rates but did not improve survival [133]. The absence of additional survival benefit likely reflects near-maximal MAPK pathway suppression with dual BRAF–EGFR inhibition, while increased toxicity with the triplet regimen may have limited treatment intensity. Consequently, the doublet regimen has been adopted as the preferred standard due to its balance of efficacy and tolerability [133,134,135,136].

Subgroup analyses demonstrated consistent benefit across clinically relevant populations, including patients with right-sided tumors, advanced age, and high metastatic burden—features frequently associated with BRAF-mutant CRC biology [7,135,137]. However, patients with poor performance status were underrepresented in pivotal trials, which may limit generalizability to some real-world populations.

Subsequent analyses and regulatory guidance have confirmed encorafenib plus cetuximab as the standard therapy for previously treated BRAF V600E–mutant mCRC [138]. Real-world studies and expanded-access programs have reported outcomes broadly consistent with trial results, although these observational data should be interpreted cautiously because of potential selection bias and heterogeneity in treatment sequencing [139,140,141].

Recent studies have explored the potential role of encorafenib-based combinations earlier in the treatment course. Early investigations suggested that targeted therapy combinations may be active in untreated disease [142]. More definitive evidence emerged from the phase III BREAKWATER trial, which demonstrated improved survival with the combination of FOLFOX, encorafenib, and cetuximab compared with chemotherapy alone, suggesting that earlier integration of targeted therapy may substantially alter outcomes in BRAF-mutant mCRC [134,143].

The safety profile of encorafenib reflects both its targeted mechanism and its use in combination regimens. Common adverse events include fatigue, nausea, diarrhea, abdominal pain, arthralgia, and dermatologic reactions, most of which are manageable with supportive care or dose adjustment [25,133,134]. Compared with earlier BRAF inhibitors, encorafenib appears to be associated with lower rates of paradoxical MAPK activation and secondary cutaneous malignancies [144,145]. When combined with EGFR inhibitors, overlapping toxicities such as acneiform rash and gastrointestinal symptoms require proactive management but are generally less severe than those associated with cytotoxic chemotherapy [104].

Despite its clinical impact, several limitations remain. Survival improvement in refractory disease remains modest, and optimal sequencing relative to immunotherapy in MSI-H/dMMR BRAF-mutant tumors remains uncertain. In addition, acquired resistance mechanisms—including MAPK reactivation through KRAS mutations, EGFR upregulation, or activation of parallel pathways—highlight the need for ongoing molecular monitoring and development of additional combination strategies [146].

Current international guidelines recommend routine BRAF mutation testing at the time of metastatic CRC diagnosis to identify patients eligible for encorafenib-based therapy [147,148,149]. Ongoing trials evaluating encorafenib combinations in earlier treatment lines and alongside immunotherapy may further refine its role within CRC treatment algorithms [150,151,152,153].

Beyond its clinical application, encorafenib has advanced the understanding of targeted therapy in CRC by demonstrating that effective pathway inhibition requires simultaneous blockade of adaptive resistance mechanisms rather than single-agent targeting [154,155,156]. It therefore represents a paradigm of rational combination therapy and molecular stratification in a historically high-risk CRC subtype [157,158,159].

Table 3 summarizes treatment-emergent adverse events and management strategies for encorafenib in CRC, and Table 4 outlines major pivotal clinical trials in mCRC.

## 4. Tucatinib

Tucatinib is an orally administered, highly selective small-molecule tyrosine kinase inhibitor (TKI) targeting the intracellular kinase domain of human epidermal growth factor receptor 2 (HER2/ERBB2) [163]. Although HER2 is a well-established oncogenic driver in breast and gastric cancers, its relevance in CRC has become increasingly apparent with advances in molecular profiling and targeted therapy development [18,164,165]. HER2-positive CRC—defined by gene amplification and/or protein overexpression—represents a distinct molecular subtype accounting for approximately 2–5% of metastatic CRC (mCRC) and is characterized by unique biological behavior, resistance patterns, and therapeutic vulnerabilities [29,166,167].

HER2 is a member of the ERBB receptor tyrosine kinase family, which includes EGFR (ERBB1), ERBB3, and ERBB4. Unlike other family members, HER2 lacks a direct ligand and functions primarily as a preferred dimerization partner, forming potent signaling complexes. Activation of HER2-containing dimers stimulates downstream oncogenic pathways, particularly the PI3K–AKT–mTOR and RAS–RAF–MEK–ERK (MAPK) cascades, promoting proliferation, survival, invasion, and metastasis of colorectal epithelial cells [168,169,170]. HER2 amplification or overexpression can also mediate resistance to anti-EGFR therapy in RAS wild-type CRC, providing the biological rationale for HER2-targeted treatment strategies [10,171,172].

**Figure 4:**
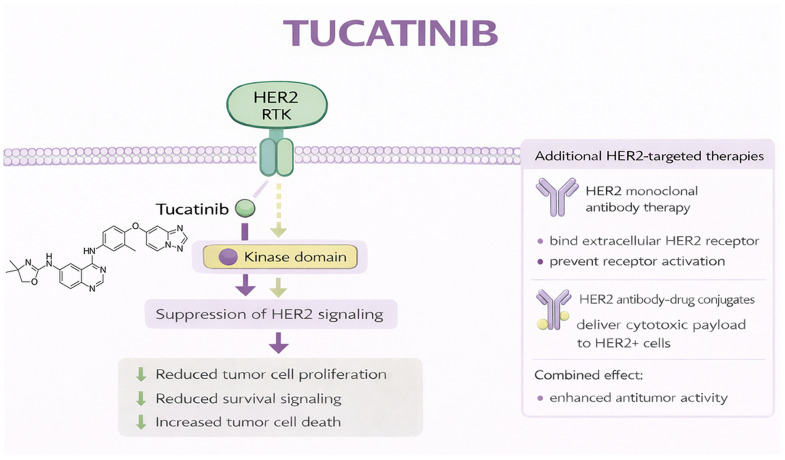
Mechanism of action of tucatinib. Original schematic illustration created by the authors based on the mechanism described in [177]. All abbreviations employed are defined in the text in the Abbreviations section.

Tucatinib was developed as a highly selective HER2 inhibitor designed to minimize off-target EGFR inhibition, thereby reducing dermatologic and gastrointestinal toxicities associated with less selective HER-family TKIs [173,174]. By binding the ATP-binding pocket of the HER2 kinase domain, tucatinib inhibits receptor autophosphorylation and downstream signaling while largely sparing EGFR-mediated physiological processes in normal tissues [173,174,175]. Preclinical studies demonstrated that maximal antitumor activity occurs when tucatinib is combined with trastuzumab, as dual HER2 blockade enhances pathway suppression and promotes tumor cell apoptosis [176]. Figure 4 presents the mechanism of action of tucatinib.

Clinical development in CRC has therefore focused on dual HER2 inhibition strategies. Evidence from several studies established that trastuzumab-based combinations can produce clinically meaningful responses in HER2-positive mCRC that has progressed after standard therapies [178,179]. The pivotal MOUNTAINEER trial provided key clinical validation of this approach, demonstrating durable responses and disease control with tucatinib plus trastuzumab in previously treated HER2-positive, RAS wild-type mCRC [31,33,180]. These findings confirmed HER2 amplification as a therapeutically actionable driver in a subset of CRC and led to regulatory approval of tucatinib in combination with trastuzumab for this indication [33].

Although the activity observed in MOUNTAINEER was clinically meaningful, interpretation of the results should consider several limitations, including the relatively modest sample size, partial single-arm design, and limited long-term follow-up [31]. Furthermore, mechanisms of acquired resistance—such as HER2 kinase domain mutations, activation of parallel pathways (e.g., MET amplification), or downstream MAPK reactivation—may limit response durability. The effectiveness of HER2-targeted therapy in HER2-mutant but non-amplified CRC also remains uncertain.

The clinical success of tucatinib underscores the importance of systematic HER2 testing in mCRC. Current ESMO and NCCN guidelines recommend evaluation of HER2 amplification or overexpression using immunohistochemistry, in situ hybridization, or next-generation sequencing, particularly in patients with RAS wild-type tumors refractory to anti-EGFR therapy [13,14,30,65]. Accurate molecular characterization is therefore essential for identifying patients who may benefit from HER2-directed treatment.

Ongoing research is exploring whether HER2-targeted therapy may be beneficial earlier in the treatment course. The MOUNTAINEER-03 trial is evaluating tucatinib plus trastuzumab combined with mFOLFOX6 as first-line therapy compared with standard chemotherapy regimens [181,182]. Additional data from studies such as the SGNTUC basket trial support the activity and manageable safety of dual HER2 blockade across HER2-altered solid tumors, although these results are not specific to CRC and should be interpreted cautiously [183,184].

Tucatinib-based therapy is generally well tolerated. The most common adverse events include diarrhea, fatigue, nausea, vomiting, and transient elevations of liver enzymes, which are typically low grade and manageable with supportive care or dose modification [31,33,180,185]. Due to its high HER2 selectivity, tucatinib is associated with lower rates of severe diarrhea and dermatologic toxicity compared with earlier HER2-targeted TKIs [173,185]. When combined with trastuzumab, cardiotoxicity remains uncommon but requires routine monitoring according to established HER2-targeted therapy guidelines [186].

Within treatment sequencing, tucatinib plus trastuzumab provides a clinically meaningful option for patients with HER2-positive mCRC who have progressed after standard chemotherapy [181]. Alternative HER2-targeted approaches such as trastuzumab deruxtecan have demonstrated higher response rates but carry a distinct toxicity profile, particularly the risk of interstitial lung disease (ILD) [178]. In the absence of direct comparative trials, treatment selection should consider disease tempo, prior therapy, and safety considerations.

Overall, tucatinib has established a defined role as targeted therapy for HER2-positive, RAS wild-type mCRC after progression on standard treatment. Ongoing trials evaluating tucatinib in earlier treatment lines, in combination with antibody–drug conjugates, and alongside immunotherapy may further refine its position within CRC treatment algorithms [165,187,188,189,190] and clarify optimal sequencing strategies [191,192].

Beyond its clinical application, tucatinib highlights the importance of identifying less common but biologically dominant oncogenic drivers in CRC. Its development illustrates how molecular characterization and rational drug design can translate into effective targeted therapies for selected patient populations, advancing the paradigm of precision oncology in CRC [158,193,194,195,196,197].

Table 5 summarizes treatment-emergent adverse events and their management in CRC, and Table 6 outlines major pivotal clinical trials of tucatinib in mCRC.

## 5. Future Directions and Contemporary Management of Metastatic Colorectal Cancer

CRC remains a major global health burden despite advances in prevention, diagnosis, and treatment. Management has evolved from stage-based algorithms to complex, multimodal, and molecularly informed strategies [202,203]. Although survival—particularly in metastatic disease—has improved, important limitations persist, including biological resistance, cumulative toxicity, inequitable access, rising costs, and uncertainty regarding optimal sequencing. Further progress will require not only therapeutic innovation but also structural and clinical refinement of current approaches [204,205].

In localized disease, surgical resection remains the cornerstone of curative therapy, often combined with perioperative or adjuvant chemotherapy based on pathologic risk. These strategies reduce recurrence and improve survival but are associated with morbidity, including bowel dysfunction, neuropathy, and long-term quality-of-life impairment [206,207]. In rectal cancer, multimodal treatment incorporating chemoradiotherapy and total mesorectal excision achieves excellent local control; however, radiation-related toxicity and functional sequelae continue to drive interest in de-escalation and non-operative strategies for selected patients [208,209]. These issues underscore the balance between oncologic efficacy and treatment-related harm in early-stage disease.

In mCRC, systemic therapy has expanded substantially. Cytotoxic chemotherapy remains foundational, but integration with targeted agents—including anti-EGFR antibodies, anti-VEGF therapies, BRAF inhibitors, HER2-directed treatments, and immune checkpoint inhibitors—has reshaped outcomes in biologically defined subgroups [65,134,181]. Molecular stratification based on RAS, BRAF, HER2, and mismatch repair status has established mCRC as a model of precision oncology. However, increasing therapeutic complexity has not been matched by clear guidance on optimal sequencing, duration, and combinations in routine practice [210,211,212].

A central limitation in advanced CRC is the near-universal development of therapeutic resistance, driven by secondary genomic alterations, pathway redundancy, tumor heterogeneity, and adaptive signaling feedback [213]. Cross-resistance among biologic agents within shared pathways further complicates sequencing. Primary and acquired resistance to anti-EGFR monoclonal antibodies represents a major challenge in extended RAS wild-type mCRC. The clinical benefit of cetuximab and panitumumab was established in trials such as PRIME and PEAK [19,78], but many patients exhibit intrinsic resistance, and nearly all responders ultimately progress. Resistance commonly arises through reactivation of MAPK signaling, including emergent KRAS, NRAS, and BRAF mutations at progression [41,214,215]. EGFR extracellular domain mutations impair antibody binding [85], while HER2 amplification, MET amplification, PIK3CA mutations, and PTEN loss restore downstream ERK signaling independently of EGFR blockade [10,39,122].

BRAF V600E–mutant mCRC is characterized by aggressive biology and intrinsic resistance to single-agent BRAF inhibition due to rapid EGFR-mediated MAPK reactivation [216]. The BEACON CRC study established encorafenib plus cetuximab as standard therapy after prior treatment [133]. The triplet regimen adding binimetinib did not significantly improve OS compared with the doublet and increased toxicity, suggesting diminishing returns from intensified vertical MAPK inhibition [133]. Future strategies include ERK inhibition, PI3K pathway combinations, and integration with immune checkpoint blockade [25,217]. In MSI-H/BRAF V600E tumors, immune checkpoint inhibitors remain standard, with combination approaches under investigation [218].

HER2 amplification defines a distinct subset of RAS wild-type mCRC. Early dual HER2 blockade demonstrated activity in refractory disease [29,30]. Antibody–drug conjugates have advanced this field; trastuzumab deruxtecan showed clinically meaningful responses in heavily pretreated HER2-positive mCRC in DESTINY-CRC01 [178]. Its mechanism couples HER2 targeting with intracellular delivery of a topoisomerase I inhibitor payload, potentially overcoming prior resistance, though interstitial lung disease (ILD) requires careful monitoring [178]. The MOUNTAINEER trial established tucatinib plus trastuzumab as an effective regimen with favorable tolerability [31]. Direct comparative data between tucatinib-based therapy and trastuzumab deruxtecan are lacking; sequencing decisions should consider disease kinetics, prior HER2 exposure, comorbidities, and pulmonary risk tolerance.

Emerging molecular monitoring strategies may help address therapeutic resistance. CtDNA and other liquid biopsy platforms offer promise for real-time resistance monitoring, yet their integration into routine decision-making remains inconsistent [219]. Longitudinal ctDNA studies demonstrate that resistant RAS-mutant clones decay after anti-EGFR withdrawal, providing a biologic rationale for rechallenge strategies [220,221]. Prospective trials such as CRICKET and CHRONOS showed that ctDNA-confirmed RAS/BRAF wild-type status at progression can identify patients who may benefit from rechallenge, reflecting a dynamic model of clonal evolution rather than static baseline genotyping [220,221]. Response rates of approximately 20–30% have been reported in selected patients, and ongoing randomized trials are refining timing and integration into later-line strategies [220,221].

Immunotherapy is standard first-line treatment for MSI-H/dMMR mCRC [39]. In MSI-H/BRAF V600E tumors, optimal sequencing between checkpoint inhibition and BRAF-targeted therapy remains uncertain. In microsatellite-stable (MSS) mCRC, immunotherapy has shown limited efficacy [218], though oncogene-targeted therapies may modulate the tumor microenvironment and enhance immune responsiveness. Trials are evaluating combinations of BRAF/EGFR inhibition with checkpoint inhibitors in MSI-H disease [25], and EGFR inhibition may enhance antigen presentation and antibody-dependent cellular cytotoxicity (ADCC), supporting combinatorial approaches in MSS tumors [222].

Optimal sequencing of anti-EGFR, anti-VEGF, BRAF-targeted, and HER2-directed therapies remains unresolved. The choice between anti-EGFR and anti-VEGF therapy in first-line extended RAS wild-type left-sided tumors is informed by FIRE-3, CALGB/SWOG 80405, and PEAK [70,71,77], though cross-trial comparisons are limited by heterogeneity. The timing of BRAF-targeted therapy relative to chemotherapy in BRAF V600E tumors remains under study [133,223,224], and prospective comparative data guiding HER2-targeted sequencing are lacking [31,178].

Several unresolved questions limit optimal care. Ideal sequencing and combinations across treatment lines remain undefined, and trial populations often fail to reflect real-world heterogeneity [210,225]. The role of treatment de-escalation after deep response, optimal therapy duration in metastatic and adjuvant settings, and validation of emerging biomarkers—including tumor microenvironment features, epigenetic changes, and microbiome composition—require further investigation [226,227].

Future progress will depend on expanded molecular profiling, including longitudinal liquid biopsy to guide adaptive strategies and detect resistance early [214,228,229]. Rational sequencing and biologically informed combinations should replace empirical escalation to maximize efficacy and limit overlapping toxicity [229,230]. Addressing tumor heterogeneity, lineage plasticity, and stromal–immune interactions is critical to overcoming treatment failure in advanced disease [213,231].

Treatment-related toxicity remains a major challenge. Chemotherapy is associated with cumulative neuropathy, myelosuppression, and gastrointestinal toxicity. Targeted therapies introduce distinct adverse-event profiles—including dermatologic reactions, hypertension, thromboembolic events, and metabolic disturbances—that may impair adherence and quality of life [232,233]. Immunotherapy, while transformative in mismatch repair-deficient CRC, carries risks of severe or irreversible immune-related adverse events [234,235,236]. As survival improves, cumulative treatment burden increasingly shapes patient experience, emphasizing toxicity mitigation and survivorship-focused care.

Cost-effectiveness, biomarker accessibility, and real-world feasibility must accompany efficacy considerations [223,224]. Access disparities persist, particularly regarding comprehensive next-generation sequencing (NGS) required to identify RAS, BRAF, HER2, and other actionable alterations [237,238]. Limited availability and delayed turnaround times may impede optimal selection, especially in community and low-resource settings [237]. Financial toxicity from combination targeted regimens and prolonged therapy further affects adherence, outcomes, and equity [239,240].

Clinical trial populations often underrepresent older adults, patients with poor performance status, and those with significant comorbidities [241,242]. Consequently, real-world toxicity and effectiveness may differ from registration studies. Contemporary mCRC management must therefore integrate molecular efficacy with feasibility, sustainability, and health-system constraints [241,242].

Accessibility and cost are central determinants of outcome. Novel agents and molecular diagnostics impose substantial financial burden, raising concerns about affordability and sustainability [243,244,245,246,247,248]. Disparities in genomic testing, multidisciplinary care, and advanced therapeutics contribute to unequal outcomes globally, particularly in low- and middle-income regions where CRC mortality is highest [249,250,251]. In resource-constrained settings, stepwise molecular testing prioritizing RAS and BRAF analysis before broader NGS panels may improve cost-effectiveness [69]. In elderly or frail patients, treatment decisions must balance toxicity risk, comorbidity burden, and expected survival benefit [13]. For BRAF V600E–mutated mCRC, encorafenib-based therapy provides a chemotherapy-sparing option with demonstrated survival benefit, though supportive and geriatric assessment remain essential [133].

Equally important are strategies to reduce treatment burden and improve quality of life through optimized dosing, integrated supportive care, and survivorship planning. Health-system research must incorporate value-based and cost-effectiveness frameworks to ensure sustainable innovation [243,250]. Moving effective targeted and immunotherapeutic approaches into earlier-stage and oligometastatic disease is under active investigation, aiming to improve cure rates rather than merely prolong survival [39,252,253,254].

Contemporary CRC management reflects substantial scientific progress but remains constrained by resistance, toxicity, access disparities, and unresolved clinical questions. Future advances must emphasize precision medicine, rational therapy integration, toxicity mitigation, and equitable access to translate innovation into durable survival and meaningful quality-of-life gains.

**Figure 5:**
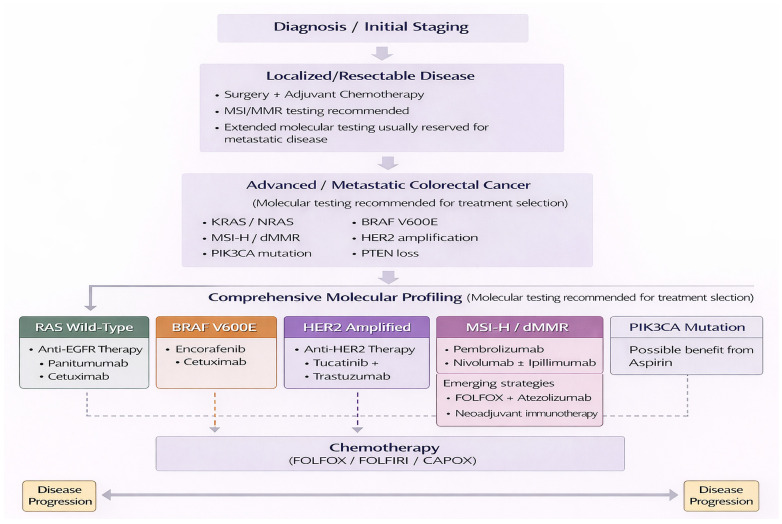
Therapeutic pathways in metastatic colorectal cancer integrating biomarkers and treatment sequencing according to [31,133,260]; original schematic illustration created by the authors. All abbreviations employed are defined in the text in the Abbreviations section.

Table 7 summarizes current management approaches cross different stages in CRC, and Figure 5 outlines a biomarker-driven treatment algorithm integrating molecular profiling with clinical staging to guide therapeutic sequencing in mCRC.

## 6. Conclusions

The clinical integration of molecularly targeted therapies against EGFR, BRAF, and HER2 has firmly established precision oncology as a central pillar of CRC management. Beyond the individual successes of agents such as panitumumab, encorafenib, and tucatinib, their collective development has reshaped how CRC is conceptualized, studied, and treated—shifting therapeutic decision-making toward molecular dependencies rather than anatomical origin alone.

These advances also make clear that effective targeting in CRC requires more than identifying a single oncogenic alteration. The colorectal tumor context—marked by pathway redundancy, adaptive feedback signaling, and pronounced intratumoral heterogeneity—often necessitates rational combinations, thoughtful sequencing, and ongoing molecular reassessment. Experience across EGFR-, BRAF-, and HER2-driven disease highlights the need to move beyond static, one-time biomarker testing toward dynamic care models that account for clonal evolution and treatment-induced resistance.

At the same time, therapeutic progress is inseparable from broader clinical and system-level considerations. Cumulative toxicity, quality-of-life trade-offs, limited durability of benefit, and substantial financial burden increasingly shape real-world outcomes and can constrain the use of otherwise effective therapies. These challenges underscore that innovation must be paired with rigorous evaluation of value, accessibility, and patient-centered outcomes, particularly as treatment options expand and survival improves.

Looking ahead, progress in molecular targeting will depend on deeper biological integration rather than incremental drug addition. Key priorities include refining predictive biomarkers beyond single-gene alterations, embedding longitudinal molecular monitoring into routine practice, and designing trials that directly address sequencing, de-escalation, and earlier intervention. Equally important is improving global equity in access to molecular diagnostics and targeted therapies so that precision oncology is not limited to select populations or healthcare systems.

Targeting EGFR, BRAF, and HER2 provides clear proof that biologically driven therapy can improve outcomes in defined subsets of CRC. The next challenge is not validating these targets, but optimizing their use—biologically, clinically, and systemically—to extend durability, minimize harm, and ensure that progress is sustainable and broadly accessible.

## Figures and Tables

**Table 1:** TEAEs and management strategies for panitumumab according to [59,64,69,86,88,90,100,101,102,103,104,105,106]. Adverse events are graded according to the Common Terminology Criteria for Adverse Events (CTCAE), version 5.0. All abbreviations employed are defined in the text in the Abbreviations section.

TEAE	Frequency/Severity	Timing/Clinical Features	Recommended Management
Papulopustular rash	Very common (≥80%); usually G1–2	Onset 1–3 wks; face/scalp/trunk	Prophylactic skin care; oral doxycycline/minocycline (≥G2); dose adjustment (≥G3); pre-emptive STEPP strategy effective
Xerosis/fissures	Common; mild–moderate	Delayed; dry, cracked skin	Emollients; urea-based creams; topical corticosteroids if inflamed
Pruritus	Common; mild–moderate	Often associated with rash	Antihistamines; topical corticosteroids; gabapentin in refractory cases
Paronychia	Common	Usually after 1–2 months; nail fold inflammation	Antiseptic soaks; topical ± oral antibiotics
Hypomagnesemia	Common; may be severe	Cumulative; muscle cramps or arrhythmias	Monitor electrolytes; oral or IV magnesium replacement
Gastrointestinal toxicity (e.g., diarrhea)	Common; usually G1–2	Early or delayed onset	Loperamide; hydration; dose modification if severe
General systemic symptoms (e.g., fatigue)	Common; mild–moderate	Variable timing	Supportive care; evaluate contributing factors
Rare or less common events (e.g., infusion reactions, ocular toxicity)	Uncommon (<5%)	Infusion reactions during/after infusion; ocular irritation or conjunctivitis	Infusion interruption and symptomatic management if reaction occurs; lubricating eye drops and ophthalmology consultation if ocular symptoms persist

Emerging strategy: topical BRAF inhibitor cream (e.g., LUT014), phase II data show reduction in ≥Grade 2 rash severity and improved quality of life; generally well tolerated, applied to affected areas during anti-EGFR therapy; targets paradoxical MAPK pathway activation in keratinocytes, investigational; may reduce rash severity and need for systemic antibiotics; currently not standard of care—awaiting phase III confirmation. Dermatologic toxicities are correlated with treatment efficacy and should be managed proactively; these adverse events should not result in premature discontinuation of therapy.

**Table 2:** Major pivotal clinical trials of panitumumab in mCRC. All abbreviations employed are defined in the text in the Abbreviations section.

Trial	Population/Cancer Setting	Design/Combination	Key Findings	Inclusion/Eligibility Criteria
PRIME (Phase III) NCT00364013 [68]	1L WT KRAS mCRC	Panitumumab + FOLFOX4 vs. FOLFOX4	↑ PFS (10.0 vs. 8.6 mo; HR 0.80); OS trend	Untreated mCRC; WT KRAS
Study 181 (Phase III) NCT00339183 [78]	2L WT KRAS mCRC	Panitumumab + FOLFIRI vs. FOLFIRI	↑ PFS (6.7 vs. 4.9 mo; HR 0.82); ↑ ORR (36% vs. 10%); no sig OS benefit	Progressed mCRC; WT KRAS
Study 408 (Phase III) NCT00113763 [59]	Chemo-refractory mCRC	Panitumumab + BSC vs. BSC	↑ PFS (HR 0.54); ORR 10% vs. 0%; no OS diff (crossover)	Refractory mCRC; EGFR+ tumors
ASPECCT (Phase III) NCT01001377 [20]	Refractory extended RAS WT mCRC	Panitumumab vs. Cetuximab	Non-inferior OS (10.2 vs. 9.9 mo; HR 0.94); similar PFS	extended RAS WT; prior irinotecan/oxaliplatin
PEAK (Phase II) NCT00819780 [77]	1L WT RAS mCRC	Panitumumab + FOLFOX vs. Bevacizumab + FOLFOX	Longer OS (esp. left-sided); similar PFS	Untreated WT RAS mCRC
PICCOLO (Phase III) NCT00389870 [107]	Advanced CRC	Irinotecan ± Panitumumab	Benefit limited to KRAS WT; harm in KRAS mutant	Post-fluoropyrimidine; KRAS status critical
VELO (Phase II) NCT03227926 [83,108]	Later-line RAS WT mCRC	FTD/TPI ± Panitumumab	↑ PFS & ORR in ctDNA-selected RAS WT	Prior anti-EGFR benefit; ctDNA RAS WT
CITRIC/CAVE-2 GOIM (Phase II) NCT04561336 [109,110]	Later-line RAS WT mCRC	Cetuximab rechallenge vs. SOC	Activity in ctDNA RAS-clear pts	Prior anti-EGFR response; ctDNA RAS WT
PARERE (Phase II) NCT04787341 [81,111]	RAS/BRAF WT mCRC	Panitumumab rechallenge vs. SOC	↑ Disease control & PFS (molecularly selected)	Prior anti-EGFR benefit; ctDNA WT

**Table 3:** TEAEs and management strategies for encorafenib according to [101,135,160,161,162]. Adverse events are graded according to the CTCAE, version 5.0. All abbreviations employed are defined in the text in the Abbreviations section.

TEAE	Frequency/Severity	Timing/Clinical Features	Recommended Management
Fatigue/Asthenia	Very common; mostly G1–2	Early onset; multifactorial	Evaluate reversible causes; supportive care and exercise; dose interruption or reduction if persistent ≥ G2–3
Nausea ± Vomiting	Common; usually G1–2	Early; intermittent	Antiemetics; hydration; small frequent meals; hold or reduce dose if ≥G3 or dehydration occurs
Diarrhea	Common; may be ≥G3	Often early; infection should be excluded	Loperamide; hydration; laboratory or stool tests if indicated; interrupt treatment if ≥G3
Acneiform rash (EGFR-related)	Very common; variable severity	Typically 1–3 weeks; face/scalp/trunk	Pre-emptive skin care; oral doxycycline/minocycline; topical corticosteroids or antibiotics; interrupt therapy if ≥G3
Abdominal pain	Common; usually G1–2	Variable onset; evaluate red flag symptoms	Symptomatic management; imaging or laboratory evaluation if severe; interrupt therapy if unexplained or severe
Decreased appetite/Weight loss	Common; usually G1–2	Early–mid treatment course	Nutritional support; treat contributing factors; dose modification if clinically significant
Musculoskeletal symptoms (arthralgia/myalgia)	Common; mostly G1–2	Usually weeks into therapy	Analgesics; physiotherapy; hold or reduce dose if persistent ≥ G2–3
Laboratory abnormalities (e.g., hepatotoxicity: ↑AST/ALT)	Common; ≥G3 less frequent	Early–mid course; laboratory-detected	Monitor liver function tests; interrupt therapy if ≥G3; resume at reduced dose after recovery
Rare or less common serious events (e.g., hemorrhage, QT prolongation, ocular toxicity such as uveitis/iritis)	Uncommon or rare; may be clinically significant	May occur at any time; QT prolongation often asymptomatic; ocular toxicity presents with eye pain, redness, or blurred vision	Evaluate and manage according to clinical severity; ECG and electrolyte monitoring for QT prolongation; ophthalmologic consultation for ocular toxicity; interrupt therapy if severe

**Table 4:** Major pivotal clinical trials of encorafenib in mCRC. All abbreviations employed are defined in the text in the Abbreviations section.

Trial	Population/Cancer Setting	Design/Combination	Key Findings	Inclusion/Eligibility Criteria
BEACON CRC (Phase III) NCT02928224 [25,135]	Previously treated BRAF V600E mCRC	Encorafenib + cetuximab ± binimetinib vs. irinotecan/FOLFIRI + cetuximab	↑ OS (9.3 vs. 5.9 mo; HR ~0.61); ↑ ORR; better tolerability; triplet ↑ ORR but no OS gain vs. doublet	BRAF V600E mCRC; 1–2 prior lines; ECOG 0–1
BREAKWATER (Phase III) NCT04607421 [143]	1L BRAF V600E mCRC	Encorafenib + cetuximab + mFOLFOX6 vs. SOC chemo ± bevacizumab	↑ ORR and deeper responses; manageable safety; OS pending	Untreated BRAF V600E mCRC; ECOG 0–1
ANCHOR CRC (Phase II) NCT03693170 [142]	1L BRAF V600E mCRC	Encorafenib + binimetinib + cetuximab (single-arm)	Meaningful ORR/DCR vs. historical chemo; supported phase III development	Untreated BRAF V600E mCRC; ECOG 0–1
Early phase Ib/II combination studies [122,123,132]	Refractory BRAF-mut mCRC	BRAF inhibitor ± EGFR ± MEK inhibitor	BRAF mono ineffective (EGFR feedback); established need for combined BRAF–EGFR inhibition	Heavily pretreated BRAF-mut mCRC

**Table 5:** TEAEs and management strategies for tucatinib according to [31,198,199]. Adverse events are graded according to the CTCAE, version 5.0. All abbreviations employed are defined in the text in the Abbreviations section.

TEAE	Frequency/Severity	Timing/Clinical Features	Recommended Management
Diarrhea	Very common; mostly G1–2	Early onset; risk of dehydration	Loperamide; hydration; rule out infection; hold treatment ≥ G3 until ≤G1 and resume ± dose reduction
Fatigue	Common; usually G1–2	Can occur at any time; multifactorial	Assess reversible causes; supportive care; dose modification rarely required
Gastrointestinal symptoms (nausea ± vomiting)	Common; usually G1–2	Early; may reduce oral intake	Antiemetics; hydration; dietary support; hold or reduce dose if persistent ≥ G3
Rash (maculopapular or acneiform)	Common; usually G1–2	Variable onset	Emollients; topical corticosteroids or antihistamines; dose modification if ≥G3
Hematologic toxicity (e.g., anemia)	Common; mostly G1–2	May present with fatigue or dyspnea	Monitor complete blood count; evaluate for deficiencies or bleeding; supportive care
Laboratory abnormalities (e.g., hepatotoxicity: ↑ALT/AST)	Common laboratory abnormality; ≥G3 uncommon	Often asymptomatic; detected on monitoring	Baseline and periodic liver function tests (e.g., every 3 weeks); hold therapy ≥G3, resume at reduced dose; discontinue if severe or recurrent
Renal laboratory changes (creatinine increase due to transporter effect)	Common; usually low grade	Early onset; not associated with true decline in GFR	Assess hydration and nephrotoxic medications; consider alternative renal markers; dose adjustment rarely required
Neurologic symptoms (e.g., headache)	Common; mild–moderate	Intermittent	Analgesics; further evaluation if persistent or associated with neurologic signs
Rare or less common events (e.g., infusion reactions)	Uncommon	During or shortly after infusion	Interrupt infusion; symptomatic management; discontinue treatment if severe

**Table 6:** Major pivotal clinical trials of tucatinib in mCRC. All abbreviations employed are defined in the text in the Abbreviations section.

Trial	Population/Cancer Setting	Design/Combination	Key Findings	Inclusion/Eligibility Criteria
MOUNTAINEER (Phase II) NCT03043313 [31,32,33]	Chemo-refractory HER2+ RAS WT mCRC	Tucatinib + trastuzumab (chemo-free)	Meaningful confirmed ORR with durable responses; basis for FDA accelerated approval	HER2+ RAS WT mCRC; prior fluoropyrimidine/oxaliplatin/irinotecan; measurable disease; ECOG per protocol
MOUNTAINEER-03 (Phase III) NCT05253651 [181,200]	1L HER2+ RAS WT unresectable/mCRC	Tucatinib + trastuzumab + mFOLFOX6 vs. mFOLFOX6 + bevacizumab or cetuximab	Primary endpoint PFS; key secondary OS & ORR; ongoing	Untreated HER2+ RAS WT unresectable/mCRC; stratified by sidedness/liver mets; ECOG per protocol
SGNTUC (Phase II, CRC cohort) NCT04579380 [183,201]	Previously treated HER2-altered solid tumors (incl. CRC)	Tucatinib + trastuzumab (basket)	Supportive evidence of dual HER2 blockade activity; non-registrational in CRC	HER2-altered advanced solid tumors; prior therapy per cohort; measurable disease; ECOG per protocol

**Table 7:** Contemporary management of CRC. All abbreviations employed are defined in the text in the Abbreviations section.

Modality	Indication/When Used	Example Regimens/Agents	Key Evidence	Biomarkers/Toxicity
Surgery	Curative treatment for localized and resectable disease; cytoreductive surgery in selected metastatic cases	Segmental colectomy, total mesorectal excision (TME), minimally invasive surgery	Surgical resection remains the primary curative modality for CRC. Advances such as total mesorectal excision and minimally invasive techniques have reduced local recurrence and improved OS, particularly when integrated into a multidisciplinary treatment approach [255]	Surgical morbidity, anastomotic leakage risk; no predictive biomarkers
Radiotherapy	Neoadjuvant or adjuvant treatment for locally advanced rectal cancer; palliation	Short-course RT, long-course chemoradiotherapy	Preoperative radiotherapy has demonstrated efficacy in tumor downstaging, improved resectability, and reduced local recurrence in rectal cancer. Both short-course and long-course regimens are effective, with treatment selection guided by tumor stage and patient characteristics [256]	Bowel dysfunction, radiation proctitis; variability in radiosensitivity
Conventional Chemotherapy	Adjuvant therapy for stage III and high-risk stage II disease; first-line treatment in metastatic CRC	FOLFOX, FOLFIRI, CAPOX, 5-FU/leucovorin	Cytotoxic chemotherapy significantly improves disease-free and OS in adjuvant and mCRC. Oxaliplatin- and irinotecan-based regimens remain standards of care despite notable toxicity profiles [257]	Neuropathy (oxaliplatin), diarrhea, myelosuppression; limited biomarker guidance
Targeted Therapy	Metastatic CRC with defined molecular alterations	Bevacizumab, Cetuximab, Panitumumab, Regorafenib	Targeted therapies have improved PFS when combined with chemotherapy. Anti-EGFR agents are effective only in RAS wild-type tumors, emphasizing the importance of molecular profiling in treatment selection [258]	Biomarkers: KRAS/NRAS/BRAF; Toxicity: skin rash, hypertension, cardiotoxicity
Immunotherapy	Advanced or metastatic MSI-H/dMMR CRC	Pembrolizumab, Nivolumab ± Ipilimumab	Immune checkpoint inhibitors produce durable clinical responses and superior survival compared with conventional chemotherapy in MSI-H/dMMR CRC, representing a conceptual transformation in the management of molecularly selected patients [256]	Biomarker: MSI-H/dMMR; Immune-related adverse events
Regional Therapy (HIPEC)	Peritoneal metastases in carefully selected patients	Cytoreductive surgery + Mitomycin C or Oxaliplatin (HIPEC)	Cytoreductive surgery combined with HIPEC may improve survival and local disease control in patients with peritoneal metastases, although benefits are confined to selected populations and associated with substantial perioperative morbidity [255]	High perioperative morbidity; lack of validated biomarkers
Emerging/Experimental Approaches	Refractory disease or clinical trial settings	Nanoparticle delivery systems, telomerase inhibitors, combination strategies	Emerging therapies targeting multidrug resistance mechanisms and tumor-specific pathways show promise in preclinical and early clinical studies, with potential for enhanced drug delivery and reduced systemic toxicity [259]	Biomarkers under investigation; toxicity varies by modality

## Data Availability

No new data were created or analyzed in this study. Data sharing is not applicable to this article.

## Abbreviations

The following abbreviations are used in this manuscript:

5-FU	5-Fluorouracil
1L	First-line
2L	Second-line
ADC	Antibody–Drug Conjugate
ADCC	Antibody-Dependent Cellular Cytotoxicity
AKT1	AKT serine/threonine kinase 1
ALT	Alanine aminotransferase
AST	Aspartate aminotransferase
BCL-XL	B-cell lymphoma extra-large
BEACON	Encorafenib plus cetuximab ± binimetinib trial
BRAF	B-Raf proto-oncogene serine/threonine kinase
BREAKWATER	Encorafenib plus cetuximab ± chemotherapy trial
BSC	Best supportive care
CALGB	Cancer and Leukemia Group B
CAPOX	Capecitabine plus oxaliplatin
CBC	Complete blood count
CCND1	Cyclin D1
CDKN1A	Cyclin-dependent kinase inhibitor 1A
CITRIC	Cetuximab rechallenge trial
CRC	Colorectal cancer
CRAF (RAF1)	C-rapidly accelerated fibrosarcoma kinase
CTCAE	Common Terminology Criteria for Adverse Events
ctDNA	Circulating tumor DNA
DAG	Diacylglycerol
dMMR	Deficient mismatch repair
ECG	Electrocardiogram
EGF	Epidermal growth factor
EGFR	Epidermal growth factor receptor
EGFR-ECD	Epidermal growth factor receptor extracellular domain
ELK1	ETS-like transcription factor 1
ERBB2 (HER2)	Erb-B2 receptor tyrosine kinase 2
ERK	Extracellular signal-regulated kinase
ESMO	European Society for Medical Oncology
FDA	U.S. Food and Drug Administration
FIRE-3	FOLFIRI plus cetuximab vs. bevacizumab trial
FOLFIRI	Fluorouracil, leucovorin, and irinotecan
FOLFOX	Fluorouracil, leucovorin, and oxaliplatin
FOLFOX4	Standard FOLFOX regimen (5-fluorouracil/leucovorin/oxaliplatin)
FTD/TPI	Trifluridine/tipiracil
G	Grade of adverse event severity (per CTCAE)
GFR	Glomerular filtration rate
GI	Gastrocolorectal
HER2	Human epidermal growth factor receptor 2
HIPEC	Hyperthermic intraperitoneal chemotherapy
HR	Hazard ratio
IV	Intravenous
JAK	Janus kinase
KRAS	Kirsten rat sarcoma viral oncogene homolog
LFT/LFTs	Liver function test(s)
MAP2K1 (MEK1)	Mitogen-activated protein kinase kinase 1
MAPK	Mitogen-activated protein kinase
mAb	Monoclonal antibody
mCRC	Metastatic colorectal cancer
MEK	MAPK/ERK kinase
MET	Mesenchymal–epithelial transition factor
MKP1	MAP kinase phosphatase-1
MMR	Mismatch repair
MSI	Microsatellite instability
MSI-H	Microsatellite instability–high
MSS	Microsatellite stable
MYC	MYC proto-oncogene
NCCN	National Comprehensive Cancer Network
NCT	ClinicalTrials.gov identifier
NGS	Next-generation sequencing
NRAS	Neuroblastoma RAS viral oncogene homolog
NSAIDs	Nonsteroidal anti-inflammatory drugs
NTRK	Neurotrophic tyrosine receptor kinase
ORR	Objective response rate
OS	Overall survival
PARADIGM	Panitumumab vs. bevacizumab trial in RAS wild-type metastatic colorectal cancer
PARERE	Panitumumab rechallenge trial
PD-1	Programmed cell death protein 1
PD-L1	Programmed death-ligand 1
PFS	Progression-free survival
PI3K	Phosphoinositide 3-kinase
PIK3CA	Phosphatidylinositol-4,5-bisphosphate 3-kinase catalytic subunit alpha
PKC	Protein kinase C
PLCγ	Phospholipase C gamma
POLE	DNA polymerase epsilon
PTEN	Phosphatase and tensin homolog
pMMR	Proficient mismatch repair
q2w	Every 2 weeks
QoL	Quality of life
QTc	Corrected QT interval
RAF	Rapidly accelerated fibrosarcoma kinase
RAS	Rat sarcoma viral oncogene family
RET	Rearranged during transfection proto-oncogene
RT	Radiotherapy
sig	Statistically significant
SOC	Standard of care
SRC	Src family kinase
STAT	Signal transducer and activator of transcription
STEPP	Skin Toxicity Evaluation Protocol with Panitumumab
T-DXd	Trastuzumab deruxtecan
TEAE	Treatment-emergent adverse event
TGF-α	Transforming growth factor alpha
TKI	Tyrosine kinase inhibitor
TME	Total mesorectal excision
V600E	Valine-to-glutamic acid substitution at codon 600
V600E/K	Valine-to-glutamic acid or lysine substitution at codon 600
VELO	Panitumumab rechallenge trial
VEGF	Vascular endothelial growth factor
WT	Wild type
